# Head‐to‐Head Comparison of Selected Extra‐ and Intracellular CO‐Releasing Molecules on Their CO‐Releasing and Anti‐Inflammatory Properties

**DOI:** 10.1002/cbic.202100452

**Published:** 2021-10-26

**Authors:** Yingchun Li, Lars Hemmersbach, Bernhard Krause, Nikolay Sitnikov, Anna Schlundt née Göderz, Diego O. Pastene Maldonado, Hans‐Günther Schmalz, Benito Yard

**Affiliations:** ^1^ Vth medical Department Medical Faculty Mannheim University of Heidelberg Theodor-Kutzer-Ufer 1–3 68167 Mannheim Germany; ^2^ Universität zu Köln Greinstrasse 4 50939 Köln Germany

**Keywords:** anti-inflammatory agents, heme oxygenases, iron carbonyl complexes, prodrugs, carbon monoxide

## Abstract

Over the past decade, a variety of carbon monoxide releasing molecules (CORMs) have been developed and tested. Some CORMs spontaneously release CO once in solution, while others require a trigger mechanism to release the bound CO from its molecular complex. The modulation of biological systems by CORMs depends largely on the spatiotemporal release of CO, which likely differs among the different types of CORMs. In spontaneously releasing CORMs, CO is released extracellularly and crosses the cell membrane to interact with intracellular targets. Other CORMs can directly release CO intracellularly, which may be a more efficient method to modulate biological systems. In the present study, we compared the efficacy of extracellular and intracellular CO‐releasing CORMs that either release CO spontaneously or require an enzymatic trigger. The efficacy of such CORMs to modulate HO‐1 and VCAM‐1 expression in TNF‐α‐stimulated human umbilical vein endothelial cells (HUVEC) was evaluated.

## Graphical abstract

**The beneficial anti‐inflammatory** effects of carbon monoxide were studied using differently triggered CO‐releasing molecules (CORMs). In particular, some acyloxy‐Fe(CO)_3_ complexes acting as enzyme‐triggered CORMs showed pronounced cellular effects regardless of whether CO release was intra‐ or extracellular.

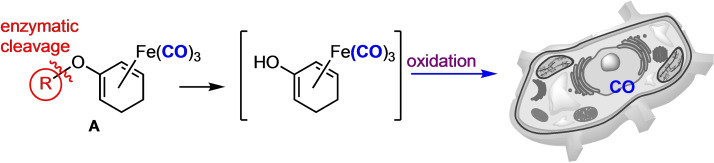

## Introduction

**Figure 1:**
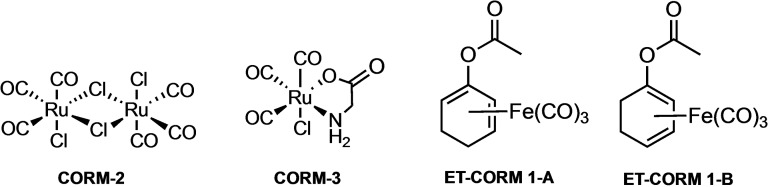
Structures of **CORM‐2**, **CORM‐3** and of **ET‐CORMs 1‐A** and **1‐B**.

**Scheme 1:**
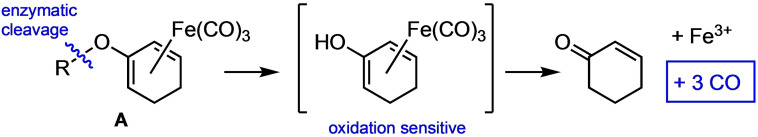
General mechanism of enzyme‐triggered CO release from ET‐CORMs of type **A**.^[6b]^

Despite its toxicity, carbon monoxide (CO) exhibits extraordinary beneficial physiological effects at low concentrations.^[1]^ Among those are anti‐inflammatory, cytoprotective, vasodilatory, anti‐bacterial and other activities,^[2]^ making CO a promising candidate for therapeutic use. However, even at low concentrations, the administration of gaseous CO bears also many risks such as headache, vomiting, loss of consciousness or even death.^[3]^ To circumvent these general toxicity problems, so‐called CO‐releasing molecules (CORMs) have been developed by several research groups to provide a more selective supply of CO into the affected tissue. First generation compounds are CORM‐2 and CORM‐3 (Figure 1) which have been used in many studies and belong to the class of spontaneous CO releasers.^[4]^ To ensure a more controllable release of CO, different types of triggered CORMs were developed.^[5]^ For instance, the so‐called photo‐CORMs release CO upon irradiation with light.[^5c^, ^5d^, ^5e^, ^5f^, ^5g^] In our own laboratory, we have developed oxy‐substituted cyclohexadiene‐Fe(CO)_3_ complexes as enzyme‐triggered CORMs (ET‐CORMS), which are equipped with esterase‐, amidase‐, protease‐ or phosphatase‐labile functionalities (Scheme 1).^[6]^

As shown in Scheme 1, these compounds are activated by the enzymatic cleavage of the R−O bond. The resulting dienol complexes are highly oxidation sensitive and disassemble under physiological conditions to release up to three equivalents of CO. In previous studies we could demonstrate that esterase‐triggered CORMs (such as **ET‐CORMs 1‐A** and **1‐B**; Figure 1) are able to selectively release CO intracellularly.[^6b^, ^6e^]

**Scheme 2:**
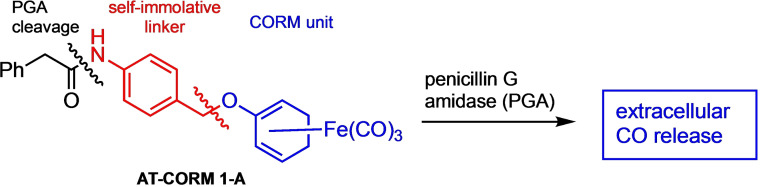
Structure and functional design of **AT‐CORM 1‐A** as an extracellular CO‐releasing molecule triggered by PGA.^[6g]^

In contrast, the so‐called amidase‐triggered **AT‐CORMs** (such as **AT‐CORM 1‐A**, Scheme 2) are triggered by penicillin G amidase (PGA), which is known to selectively cleave the phenylacetyl amide bond.^[6g]^ In a secondary step, the self‐immolative linker falls apart to generate the same oxidation‐sensitive dienol‐Fe(CO)_3_ intermediate from which CO is finally released. As externally added PGA cannot cross the cell membrane, the CO release is supposed to occur extra‐cellularly in this case.

Because spatiotemporal release of CO to biological systems likely influences its biological effects, the present study was conducted to compare the efficacy of extra‐ and intracellular CO releasing CORMs that either spontaneously release CO or require an enzymatic trigger. For this purpose, we studied the efficacy of such CORMs to induce heme oxygenase 1 (HO‐1) and to inhibit the expression of VCAM‐1 (vascular cell adhesion molecule 1) in TNF‐α stimulated human umbilical vein endothelial cells (HUVEC). These two proteins (HO‐1 and VCAM‐1) both represent key players/markers in cellular CO metabolism.[^5a^, ^5b^, ^6e^, ^6f^]

## Results

### Definition and structural characteristics of extra‐ and intra‐cellular CO‐releasing CORMs

**Figure 2:**
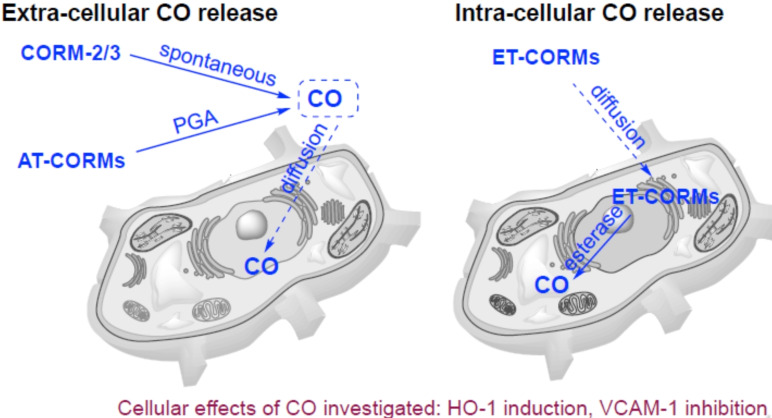
Different behavior of various types of CORMS: While **CORMs‐2**/**3** (as spontaneous CO releasers) and **AT‐CORMs** (activated by PGA) release CO in the extracellular space, **ET‐CORMs 1‐A/1‐B** are supposed to act primarily as intracellular CO releasers.

**Figure 3:**
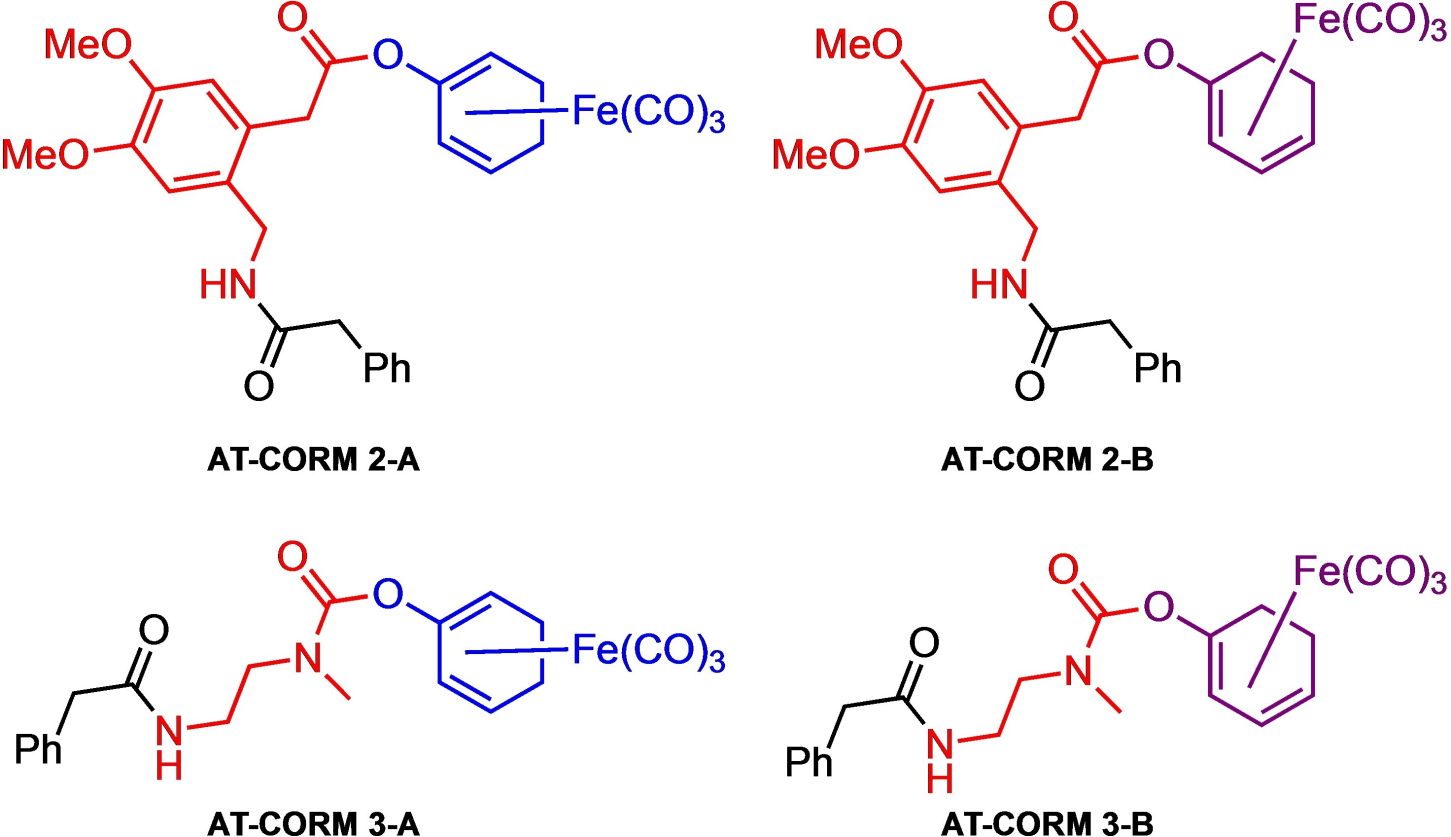
Structures of **AT‐CORM‐2‐A/B** and **AT‐CORM‐3‐A/B** as extracellular PGA‐triggered CO releasers. Note: Due to the ester function **AT‐CORMs‐2‐A/B** may additionally be activated by intracellular esterases.

In the present study we compared six different CORMs with respect to their CO releasing properties, their cytotoxicity, their ability to induce HO‐1 expression and their ability to inhibit the expression of VCAM‐1 in TNF‐α stimulated HUVEC. As already outlined above, the CORMs investigated were classified as extra‐cellular CO‐releasing CORMs, i. e. CORMs which do not require internalization to release CO, and intra‐cellular CO‐releasing CORMs of which CO release strictly depends on intra‐cellular esterase activity. The former CORMs were further sub‐classified as spontaneous releasing – and amidase‐triggered CORMs, respectively (Figure 2). For the AT‐CORMs three types of linkers were used (1) a 1,6‐benzyl elimination linker attached to the CORM unit via an ether bond^[7]^ (**AT‐CORM 1‐A**,^[6g]^ Scheme 2), (2) a cyclization linker attached via an ester bond^[8]^ (**AT‐CORM 2‐A** and **AT‐CORM 2‐B**) or (3) a cyclization linker attached to the oxydiene −Fe(CO)_3_ unit via a carbamate bond^[9]^ (**AT‐CORM 3‐A** and **AT‐CORM 3‐B** ^[6g]^). The structures of **AT‐CORMs** of type **2** and **3** are shown in Figure 3.

Noteworthy, **AT‐CORMs 2‐A** and **2‐B** not only possess a PGA‐cleavable phenylacetamide unit but also an ester function. Therefore, these compounds are potentially prone to both esterase and PGA activation.

For our present study we thus employed **CORMs‐2/3**,^[4]^ **ET‐CORMs 1‐A/B**,^[6a]^ the known **AT‐CORMs** of type **1** and **3** ^[6g]^ as well as the new type, **AT‐CORMs 2‐A** and **2‐B**, which were synthesized as detailed in the Supporting Information. One should be aware that all diene‐Fe(CO)_3_‐based CORMS were employed as racemic mixtures (due to the planar chirality of the diene‐Fe(CO)_3_ unit) and that the oxy‐substituent was either positioned at the “inner” (A series) or at the “outer” position (B series) of the 1,3‐cyclohexadiene‐Fe(CO)_3_ moiety.

### CO‐releasing properties

**Figure 4:**
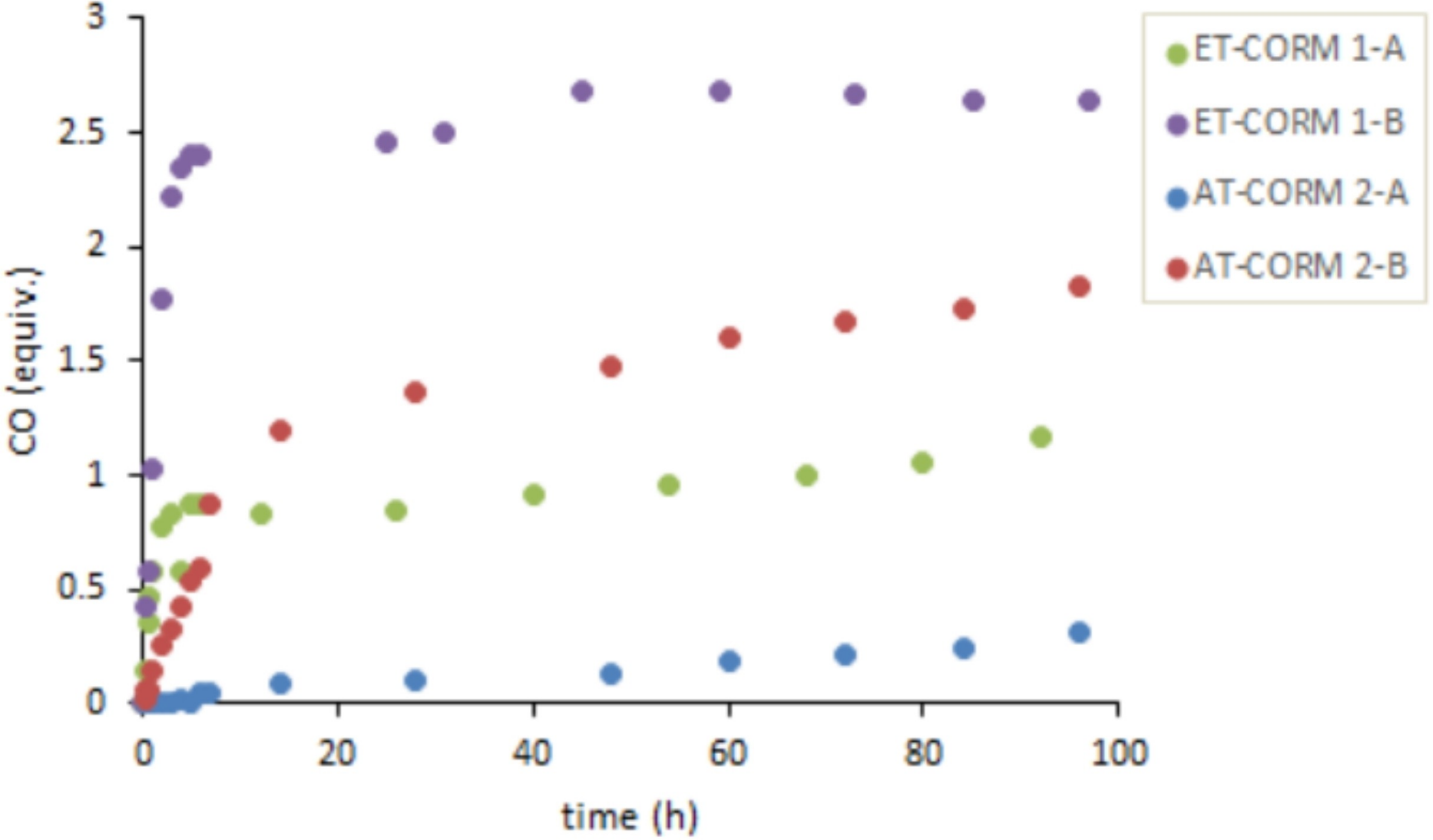
In vitro CO release from **ET‐CORM 1‐A/1‐B** and **AT‐CORM 2‐A/2‐B** in the presence of PLE. CO was detected by headspace GC; no CO release was detected in absence of PLE.

**Figure 5:**
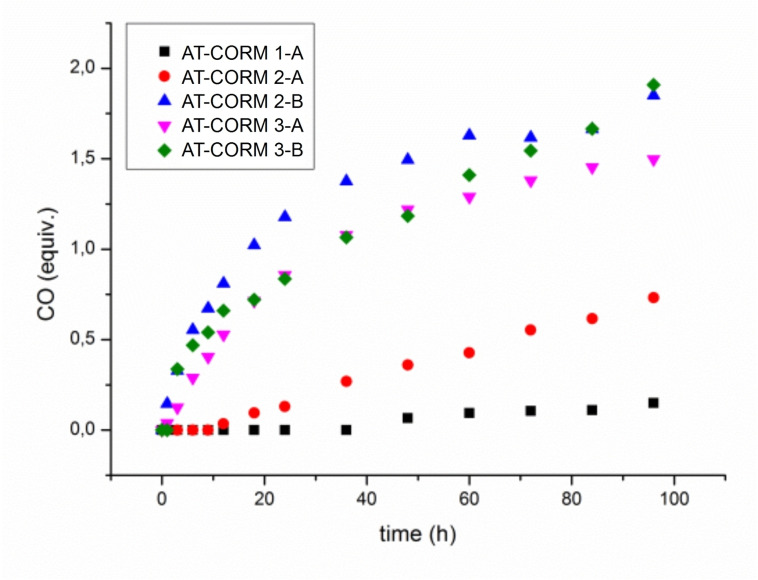
In vitro CO release from **AT‐CORMs** in the presence of PGA. CO was detected by headspace GC; no CO release was detected in absence of PGA.

**Figure 6:**
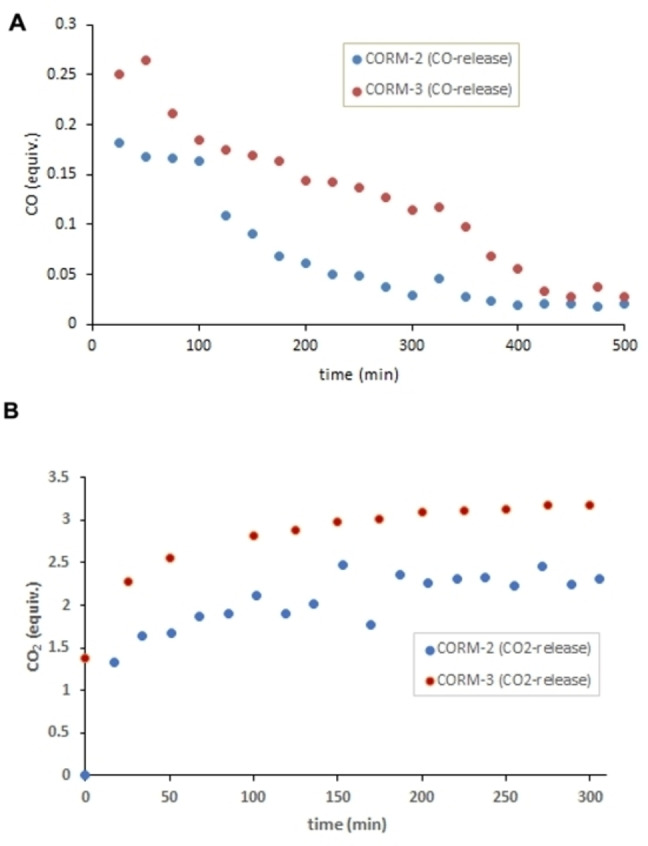
In vitro CO (**A**) and CO_2_ (**B**) release from **CORM 2 and CORM 3** in phosphate buffer (0.1 M; pH=7.4) / DMSO=5 : 1 as detected by headspace GC.

Headspace gas chromatography (GC) was used to quantify CO release in vitro using a 5 : 1 mixture of phosphate buffer (0.1 M; pH=7.4) and DMSO. In the case of triggered CO release, porcine liver esterase (PLE) or penicillin G amidase (PGA) was used in combination with **ET‐CORM 1‐A**, **ET‐CORM 1‐B**, and **AT‐CORMs**, respectively. With these compounds, enzyme‐induced CO release provided significant amounts (up to 2.5 equivalents for **ET‐CORM 1‐B**) of detectable CO (Figures 4 and 5). In contrast, only very small amounts of CO (less than 0.3 equivalents) were detected for **CORM‐2** and **CORM‐3** besides significant amounts of CO_2_. In fact, for **CORMs‐2/3**, the amount of CO_2_ (per mmol) was more than ten times higher than the amount of CO and actually increased slightly with time (Figure 6), while the CO concentration decreased, possibly as a result of Ru‐catalyzed oxidation of CO to CO_2_.^[10]^

The esterase‐triggered CORMs (**ET‐CORMs**) yielded higher CO equivalents than the corresponding amidase‐triggered **AT‐CORMs** with the same substituent position, and as a second general trend, compounds with the oxy‐substituent at the “outer” position of the cyclohexadiene‐Fe(CO)_3_ moiety (B series) were confirmed to show a faster and stronger CO release. For example, **AT‐CORM 2‐B** released less CO than **ET‐CORM 1‐B**, and **AT‐CORM 2‐A** released less CO than **ET‐CORM 1‐A** (Figure 4).

The fact that **AT‐CORMs 2 A/B** also showed CO‐release in the presence of PLE (Figure 4) indicated the sensitivity of the ester function of these compounds towards PLE as a model esterase. However, as Figure 5 shows, these compounds were efficiently triggered also by PGA to release more than 1.5 equivalents of CO within 50 hours under the standard conditions. Amongst the amide‐triggered CORMS, **AT‐CORM 2‐B**, and **AT‐CORMs 3‐A/3‐B** were the most effective (Figure 5), while very little CO was liberated from **AT‐CORM 1‐A** in accord with the results previously published for this compound.^[6g]^

As already mentioned above, only very little CO was generated (spontaneously) upon dissolution of **CORMs 2** and **3** in a phosphate buffer/DMSO mixture (Figure 6). Noteworthy, the detectable amount of CO vanished more or less completely within 7 hours, apparently at the expense of CO_2_ which is rapidly evolving in comparably large amounts within a few minutes. This observation is in agreement with a report of Romão and co‐workers who also detected significant amounts of CO_2_ formed from **CORM‐2** and **CORM‐3**.^[11]^ Also, Poole and co‐workers have recently shown that **CORM‐2** only releases negligible amounts of CO (<0.1 mol CO per mol **CORM‐2**) and concluded that the biological effects of **CORM‐2** and related CORMs should be re‐examined in the light of these data.^[12]^

### Cell toxicity

**Figure 7:**
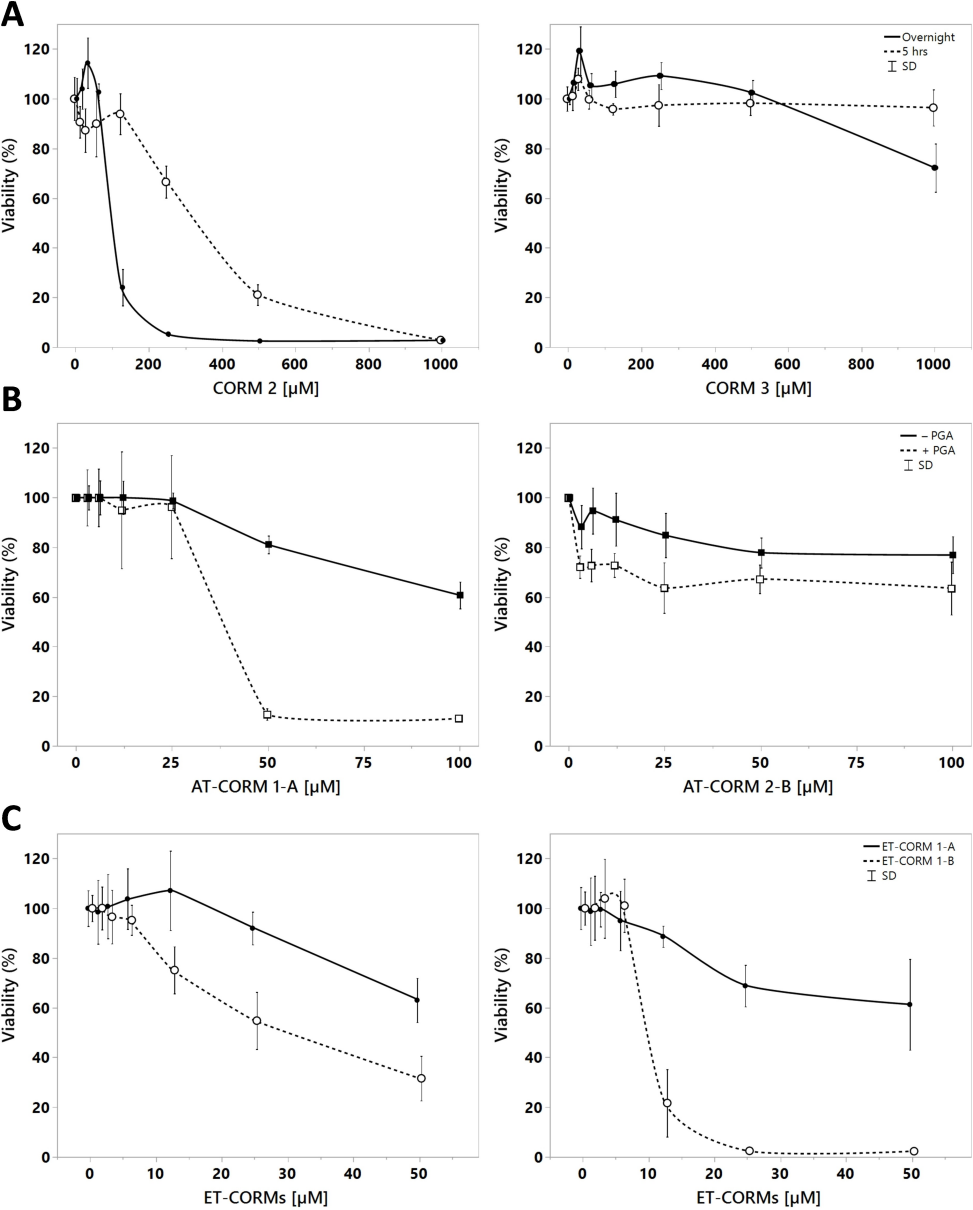
Cell viability as assessed by MTT for extracellular – (**A**) and intracellular releasing CORMs (**B** and **C**). For the spontaneous CO releasing CORMs cell viability was assessed at early (5 h, dotted line) and late (overnight, drawn line) time‐points following stimulation. For amidase triggered CO releasing CORMs viability was assessed after overnight incubation with AT‐CORMs in the presence (dotted line) or absence (drawn line) of PGA (**B**). For esterase triggered CORMs viability was assessed at two different time points (5 h: graph to the left and overnight: graph to the right, drawn line **ET‐CORM 1‐A**, dotted line **ET‐CORM 1‐B**)(**C**).

We next assessed the toxicity of the extra‐ and intra‐cellularly acting CORMs in HUVEC by means of MTT.^[13]^ For the spontaneously extra‐cellular CO‐releasing CORMs (**CORM‐2** and **CORM‐3**) toxicity occurred at higher concentrations as compared to the amidase triggered extra‐cellular CO‐releasing CORMs (in the presence of PGA) (Figure 7). With respect to the former CORMs, toxicity was only observed after overnight incubation for **CORM‐3** (cell viability approximately 75 %), while for **CORM‐2** toxicity was already evident 5 h after addition of **CORM‐2**. Endothelial cells displayed a slightly decreased cell viability after overnight incubation with the amidase‐triggered extra‐cellular CO‐releasing AT‐CORMs. Cell viability significantly decreased in the presence of PGA for **AT‐CORM 1‐A** but not for **AT‐CORM 2‐B** (cell viability after incubation with **AT‐CORM 1‐A**: 70 % vs 15 %, and with **AT‐CORM 2‐B**: 80 % vs 70 %; for no PGA vs PGA added) (Figure 7). The intra‐cellular, as compared to the extra‐cellular CO releasing CORMs, displayed in general toxicity at lower concentrations. As reported previously, **ET‐CORM 1‐B** was more toxic compared to **ET‐CORM 1‐A**.^[6f]^ This was already noticed at early time points of incubation but the difference in toxicity became more prominent at later time points (Figure 7).

### Amidase dependency of AT‐CORMs

**Figure 8:**
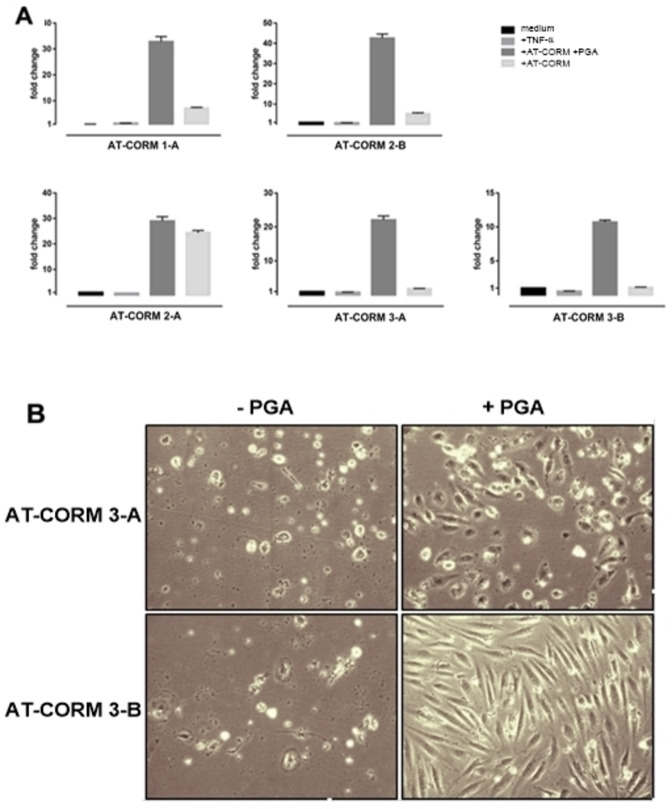
Amidase dependent induction of HO‐1 by AT‐CORMs. **A**: Unless otherwise mentioned, HUVEC were stimulated throughout all experiments for a defined period with TNF‐α (10 ng/ml) and different AT‐CORMs in the presence or absence of 1 μg/ml of penicillin‐G amidase (PGA). HUVEC kept in normal culture medium was included in each experiment. Cells were treated as described above overnight with 50 μM of AT‐CORMs. Hereafter total RNA was isolated to assess HO‐1 mRNA expression by means of quantitative PCR. The results were normalized to β‐actin and expressed as fold change relative to the untreated medium control by using the ΔΔC_t_ method. **B**: Cell morphology of HUVEC treated with 50 μM of **AT‐CORM 3‐A** or **AT‐CORM 3‐B** in the presence or absence of PGA. Note that both CORMs displayed toxicity in the absence of PGA while toxicity was ameliorated by the addition of PGA.

Since CO is a potent inducer of HO‐1 expression,^[14]^ we used HO‐1 induction as a read‐out to assess the amidase dependency of AT‐CORMs. With exception of **AT‐CORM 2‐A**, induction of HO‐1 mRNA only occurred when both penicillin G amidase (PGA) and AT‐CORMs were added to HUVEC (Figure 8). Because in **AT‐CORM 2‐A** and **AT‐CORM 2‐B** the self‐immolative linker is attached to the η^4^‐oxydiene‐Fe(CO)_3_ moiety via an ester bond, these structures are potentially also cleavable through intracellular esterase activity. Yet, only for **AT‐CORM 2‐A** PGA dependency was compromised suggesting that hydrolysis of **AT‐CORM 2‐A** by intracellular esterases is comparably fast, while **AT‐CORM 2‐B** is not or only little affected by intracellular esterases. Interestingly, this different behavior does not correspond to the in vitro experiments using PLE, which suggest a faster cleavage and a more pronounced CO release in the case of **AT‐CORM 2‐B** (Figure 4).

Although **AT‐CORM 3‐A** and **AT‐CORM 3‐B** appeared to be PGA specific, strong morphological signs of toxicity were observed already in the absence of PGA. Interestingly, cell morphology clearly improved, particularly for **AT‐CORM 3‐B**, when PGA was present (Figure 8). Since **AT‐CORM 2‐A** did not display PGA specificity for HO‐1 induction and for **AT‐CORM 3‐A** and **AT‐CORM 3‐B** the data were inconclusive, they were excluded for further comparisons.

### Comparisons of different CORMs to modulate HO‐1 and VCAM‐1 expression

**Figure 9:**
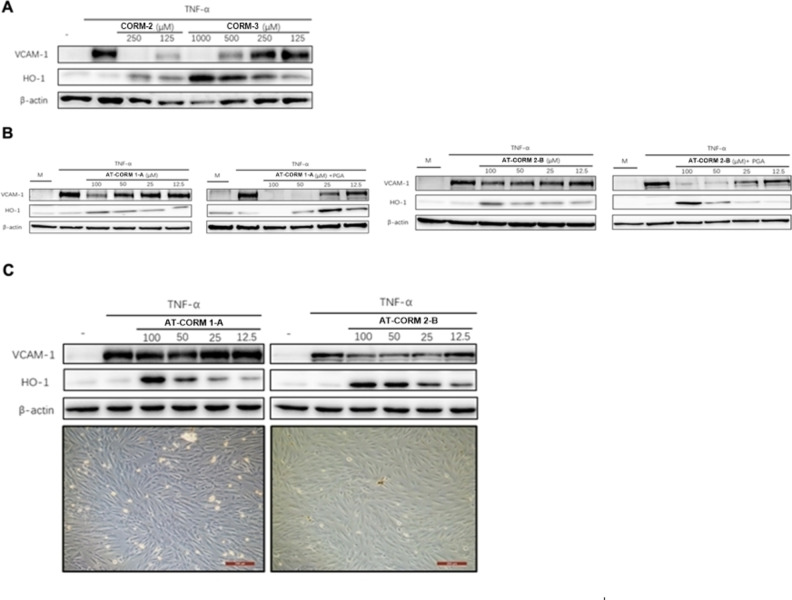
Inhibition of TNF‐α mediated VCAM‐1 expression and induction of HO‐1 by extra‐cellular CORMs. **A**: Spontaneous releasing CORMs were tested at different concentration and overnight incubation. **B**: Amidase triggered CORMs were tested at significant lower concentration (0–100 μM) either in the presence (panels to the right) or absence (panels to the left) of PGA. The cells were stimulated overnight. **C**: Since at the highest concentration toxicity was observed particularly for **AT‐CORM 1‐A**, cells were also stimulated for 5 h at which no cell morphological changes typically occurring for toxic compounds were noticed.

**Figure 10:**
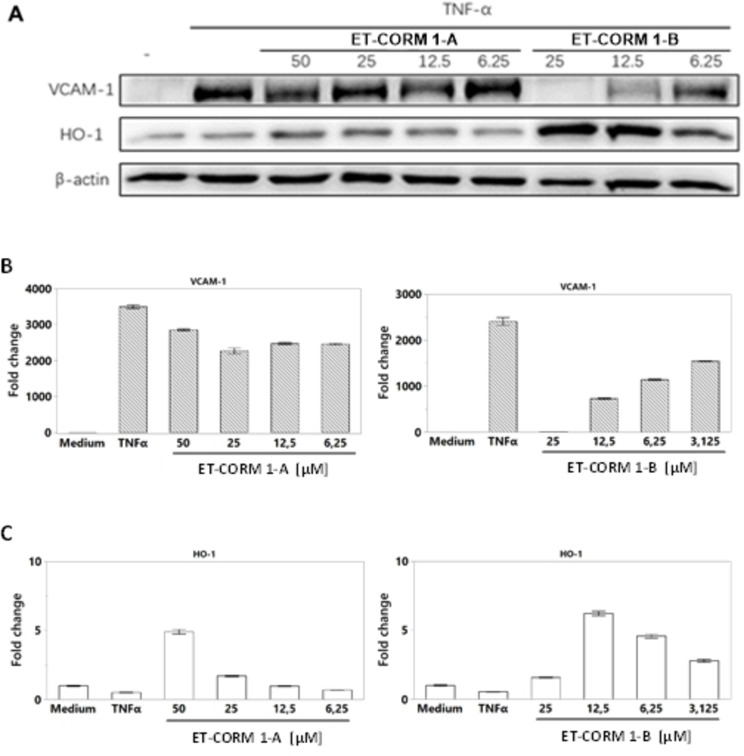
Inhibition of TNF‐α mediated VCAM‐1 expression and induction of HO‐1 by intra‐cellular CO releasing CORMs. VCAM‐1 and HO‐1 protein (**A**) and mRNA expression for VCAM‐1 (**B**) and HO‐1 (**C**) are depicted.

We next assessed the efficacy of the different CORMs to modulate HO‐1 and VCAM‐1 expression. All of the selected CORMs were able to induce HO‐1 and to inhibit TNF‐α‐mediated VCAM‐1 expression in a dose‐dependent manner. Yet, for each of the individual CORMs this occurred at different CORM concentrations. While for the spontaneously CO releasing CORMs (**CORM‐2** and **CORM‐3**) relatively high concentrations were required, for the triggered CO‐releasing CORMs modulation of HO‐1 and VCAM‐1 expression occurred at much lower concentrations (Figures 9 and 10). **CORM‐3** was slightly more efficacious compared to **CORM‐2** at comparable CORM concentrations. (Figure 9). Similar, as shown for the induction of HO‐1 mRNA (Figure 8), VCAM‐1 expression was inhibited by **AT‐CORM 1‐A** and **AT‐CORM 2‐B** in an amidase‐dependent manner. Because cell viability was compromised when HUVEC were stimulated overnight with **AT‐CORM 1‐A** at and above 50 μM, we also assessed modulation of VCAM‐1 and HO‐1 expression by **AT‐CORM 1‐A/2‐B** at an early time‐point (5 h following stimulation) at which no toxicity was observed (Figure 9). While **AT‐CORM 1‐A** did not inhibit VCAM‐1 at this time‐point, HO‐1 expression was clearly induced. For **AT‐CORM 2‐B** also at an early time‐point VCAM‐1 expression was slightly inhibited accompanied by a strong induction of HO‐1 (Figure 9).

For both the AT‐CORMs and ET‐CORMs modulation of HO‐1 and VCAM‐1 expression was more pronounced when the self‐immolative linker respectively the ester function was positioned at the “outer” position of the 1,3‐cyclohexadiene‐Fe(CO)_3_ moiety. At this position, the intra‐cellular CO‐releasing CORMs **ET‐CORM 1‐B** was slightly more efficacious compared to extra‐cellular CO‐releasing **AT‐CORM 2‐B**. In contrast, no difference was found between **ET‐CORM 1‐A** and **AT‐CORM 1‐A**. Similar as shown for the other CORMs, modulation of HO‐1 and VCAM‐1 expression by ET‐CORMs was also noticed at the transcriptional level (Figure 10).

## Discussion

In the present study we compared the efficacy of selected extra‐ and intracellular CO releasing CORMs that either spontaneously release CO or require an enzymatic trigger to modulate HO‐1 and VCAM‐1 expression. The main findings are as follows. Firstly, CO release from the spontaneously CO releasing CORMs (**CORM‐2/3**) was significantly lower as compared to the ones requiring an enzymatic trigger. Esterase‐triggered CO release was higher as compared to that triggered by amidase, provided that the oxy‐attached linker in the **AT‐CORMs** was positioned at a similar site of the 1,3‐cyclohexadiene‐Fe(CO)_3_ unit as the corresponding **ET‐CORM**. In general CO release was stronger when the oxy‐substituent was positioned at the “outer” position of the 1,3‐cyclohexadiene‐Fe(CO)_3_ moiety. Secondly, a relation between the extent of CO release and toxicity of the CORMs was observed, i. e. toxicity was the lowest for CORMs with relatively low CO release (**CORM‐2/3**) and the highest for those that released large amounts of CO (**ET‐CORM 1‐B**). Thirdly, HO‐1 induction by **AT‐CORMs** was strictly PGA dependent for **AT‐CORMs 1‐A**, **2‐B**, **3‐A** and **3‐B. AT‐CORMs 3‐A** and **3‐B** however displayed strong toxicity in the absence of PGA which might have impeded the induction of HO‐1. Finally, modulation of HO‐1 and VCAM‐1 expression occurred for all CORMs at different concentrations with the intra‐cellular CO‐releasing **ET‐CORM 1‐B** being the most efficacious.

We have also assessed CO release of the ruthenium‐ (**CORM‐2/3**) and iron‐based CORMs (**AT‐CORMs**, **ET‐CORMs**) by means of headspace‐GC. Our findings for the ruthenium‐based CORMs are in line with previous studies that revealed mainly CO_2_ – rather than CO release by these CORMs.^[11]^ For the iron‐based CORMs, triggered by esterase or amidase, no CO_2_ was detected. Although the large difference in CO release between both types of CORMs may explain why toxicity of the iron‐based CORMs was generally observed at lower concentrations, CO release it‐self cannot explain why **CORM‐2** was significantly more toxic compared to **CORM‐3**. We have previously shown that toxicity of the iron‐based CORMs is unlikely explained by the amount of iron that is released upon hydrolysis and might be a consequence of inhibition of cell respiration.^[6e]^

The anti‐inflammatory propensity of CO is well recognized and its potential therapeutic use demonstrated in many in vitro and vivo models of inflammation. The induction of HO‐1 and inhibition of VCAM‐1 expression was chosen as read‐out to study the efficacy of the selected CORMs, because in previous studies **CORM‐2/3**,^[15]^ **ET‐CORMs** ^[6f]^ and **AT‐CORMs** ^[6g]^ were all able to modulate the expression of these molecules. In line with their CO releasing properties, it was found that **CORM‐2/3** was less efficacious in modulating the expression of HO‐1 and VCAM‐1. Despite the poor CO releasing property and their moderate effect to modulate HO‐1 and VCAM‐1 expression, a number of in vivo studies have demonstrated a beneficial effect in a variety of disease models.^[16]^ This might be explained by **CORM‐2/3**‐mediated induction of HO‐1. Although this also occurred at high concentration in our study on endothelial cells, other studies have revealed that induction of HO‐1 by **CORM‐2/3** occurred at low concentrations in immune cells e. g. macrophages.^[17]^ It therefore would be prudent to take some caution in concluding that the anti‐inflammatory effect of enzymatic triggered CORMs is stronger compared to that of **CORM‐2/3**, as a limited number of inflammatory parameters in only one type of cells was investigated and thus far no in vivo studies have been performed with the former CORMs.

Since CO release of **ET‐CORM 1‐B** and **AT‐CORM 2‐B** was mediated by intra‐ and extracellular enzymes respectively mediating a relatively comparable CO release, differences in efficacy between both types of delivery could be compared. While for the anti‐inflammatory effect **ET‐CORM 1‐B** was slightly more efficacious, for toxicity the difference was more pronounced. It thus seems that intracellular CO release is more efficacious compared to extracellular CO release for CORM‐mediated toxicity, but not so much for the CORM‐mediated anti‐inflammatory properties. Our data corroborate previous studies^[18]^ that reached the conclusion that extra‐cellular CO release is less toxic while the anti‐inflammatory properties are similar to that of intra‐cellular CO release.

## Conclusion

The present study reinforces and extents previous reports on the anti‐inflammatory properties of CORMs. In essence, it shows that these effects more closely correlate with the amount of CO released from the CORM rather than with intra‐ or extra‐cellular CO delivery. It was found that the ruthenium‐based **CORM‐2** and **CORM‐3** mainly liberate CO_2_ (besides only little CO), confirming the results of Poole and co‐workers that the use of these first‐generation CORMs as reference CORMs may no longer be appropriate.^[12]^ Noteworthy, no CO_2_ was released from the iron‐based **AT‐** and **ET‐CORMs** after enzymatic hydrolysis. Based on the aforementioned observation that extra‐ and intra‐cellular CO delivery yield similar anti‐inflammatory properties, our study suggests that the use of specific membrane associated enzymatic activity may pave the way for tissue‐targeted CO delivery. Identification of such enzymes and implementation of their specificity for hydrolysis of oxy‐substituted cyclohexadiene‐Fe(CO)_3_ complexes are subject of future studies.

## Conflict of interest

The authors declare no conflict of interest.

## Supporting information

As a service to our authors and readers, this journal provides supporting information supplied by the authors. Such materials are peer reviewed and may be re‐organized for online delivery, but are not copy‐edited or typeset. Technical support issues arising from supporting information (other than missing files) should be addressed to the authors.

Supporting Information
