# Supplementary material for: Head‐to‐Head Comparison of Selected Extra‐ and Intracellular CO‐Releasing Molecules on Their CO‐Releasing and Anti‐Inflammatory Properties
Source: Chembiochem. 2021 Oct 26;23(1):e202100452. doi: 10.1002/cbic.202100452 (PMC9298253; doi:10.1002/cbic.202100452)
Supplement: Supplementary file 1 — Supporting Information [file CBIC-23-0-s001.pdf]

ChemBioChem

Supporting Information

Head-to-Head Comparison of Selected Extra- and Intracellular CO-Releasing Molecules on Their CO-Releasing and Anti-Inflammatory Properties

Yingchun Li[+], Lars Hemmersbach[+], Bernhard Krause, Nikolay Sitnikov, Anna Schlundt née Göderz, Diego O. Pastene Maldonado, Hans-Günther Schmalz,* and Benito Yard*

Table of Contents

1. General Information 1
2. Synthetic Procedures 2
   2.1. Synthesis of AT-CORM 2-A 2
   2.2. Synthesis of AT-CORM 2-B 3
3. NMR Spectra 5
4. In situ quantification of CO and CO_2 release 7
5. Biological Investigations 8
6. References 9

1. General Information

Unless otherwise specified, all reactions were carried out under inert conditions. Glassware was heat-dried under vacuum and flushed with argon (*Linde*® Argon 4.6 (99.996 %, <1 ppm water, <1 ppm oxygen). Solids were added under argon counter flow. Liquids were added with syringes through gas-tight septa. Before use, syringes were flushed with argon thrice.

Reagents were purchased from commercial suppliers (*Sigma-Aldrich*, *Lancaster*, *Alfa Aesar*, *Carbolution*, *ABCR* or *Acros*) and used without further purification.

All reactions were monitored by TLC using 0.25 mm Merck silica gel 60 F254 precoated plates and visualized with UV light (Mineralight UVGL-25 lamp) or by staining with a potassium permanganate solution (3 g $KMnO_4$, 5 mL NaOH (5 Vol.-%) in 300 mL H_2O) followed by heating.

Flash column chromatography was conducted with silica gel from *Acros* (60 Å, 0.035 nm – 0.070 nm).

^{1}H and ^{13}C NMR spectra were recorded on a *Bruker* Avance 400 (^{1}H NMR: 400 MHz; ^{13}C NMR: 100 MHz). Chemical shifts are given in ppm (parts per million). Coupling constants are reported in Hz. Multiplicities are specified using the following abbreviations: s (singlet), d (doublet), t (triplet), q (quartet), m (multiplet) and combinations of the abbreviations.

HR-ESI-MS spectra were measured on a THERMO Scientific LTQ Orbitrap XL-FTMS Analyser using electrospray ionization (3.4 kV spray voltage). The capillary and tube lens voltage were set to 3.0 V.

IR spectra were recorded at room temperature on an UATR TWO FT-IR-spectrometer from *Perkin Elmer*. Wavenumbers $\tilde{\nu}$ [cm^{-1}] and relative intensities of selected signals are listed using the following abbreviations: s (strong), m (medium), w (weak).

2. Synthetic Procedures

2.1. Synthesis of AT-CORM 2-A

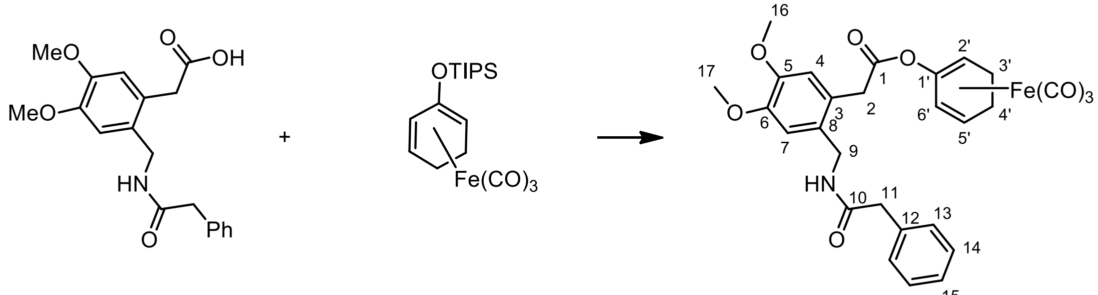

1 *rac*-**2-A** **AT-CORM 2-A**

Under argon atmosphere, a solution of TIPS-protected complex *rac*-**2-A**[1] (250 mg, 0.64 mmol, 1.0 eq.) in anhydrous DMF (5.0 mL) was cooled to 0 °C. TBAF (1 M solution in THF, 640 µL, 0.64 mmol, 1.0 eq.) was added and the reaction mixture was stirred for 10 min at 0 °C. Then pyridine (83 µL, 0.64 mmol, 1.0 eq.) and HBF_4*Et_2O (87 µL, 0.64 mmol, 1.0 eq.) were added and the mixture was stirred for further 10 min.

In a second flask, under argon atmosphere, a solution of acid **1**[2] (219 mg, 0.638 mmol, 1.0 eq.) in anhydrous DMF (5 mL) was cooled to 0 °C. Then pyridine (166 µL, 1.28 mmol, 2.0 eq.), DIC (298 µL, 1.91 mmol, 3.0 eq.), HOBt (25.8 mg, 0.191 mmol, 0.3 eq.) and DMAP (23.3 mg, 0.191 mmol, 0.3 eq.) were added. Subsequently, the dienol-complex was added, and the mixture was stirred at room temperature for 12 h.

The mixture was diluted with EtOAc (45 mL), washed with HCl (0.1 N, 2 x 20 mL), water (20 mL) and brine (20 mL). The organic phase was dried with $MgSO_4$ and the solvent was removed under reduced pressure. The residue was purified by column chromatography (SiO_2, cHex/EtOAc/$CHCl_3$ = 1:1:0.5) to yield **AT-CORM 2-A** (265 mg, 0.472 mmol, 74%) as a yellow oil.

M($C_{28}H_{27}FeNO_8$): 561.37 g mol^{-1}.

TLC: R_f (cHex/EtOAc/$CHCl_3$ = 1:1:0.5) = 0.25.

^{1}H NMR: (400 MHz, $CDCl_3$) δ = 7.35 – 7.29 (m, 2H, H-14), 7.28 – 7.24 (m, 3H, H-13, H-15), 6.74 (s, 1H, H-7), 6.69 (s, 1H, H-4), 6.05 – 5.97 (m, 1H, NH), 5.53 – 5.48 (m, 1H, H-6’), 4.47 – 4.31 (m, 2H, H-9), 3.84 (s, 3H, H-16), 3.80 (s, 3H, H-17), 3.71 (d, ^2J = 3.1 Hz, 2H, H-2), 3.58 (s, 2H, H-11), 3.30 (dt, ^3J = 3.6, ^4J = 2.4 Hz, 1H, H-2’), 2.86 (ddd, ^3J = 6.6, 3.5, 2.5 Hz, 1H, H-5’), 1.83 – 1.67 (m, 2H, H-3’), 1.65 – 1.45 (m, 2H, H-4’).

^{13}C NMR: (100 MHz, $CDCl_3$): δ = 210.7 ($Fe(CO)_3$), 171.6 (C-1), 170.8 (C-10), 148.8 (C-6), 148.6 (C-5), 135.0 (C-12), 129.5 (C-14), 129.4 (C-8), 129.1 (C-13), 128.5 (C-1'), 127.4 (C-15), 123.6 (C-3), 113.6 (C-4), 112.9 (C-7), 79.9 (C-6'), 58.9 (C-2'), 56.1 (C-16), 56.1 (C-17), 52.3 (C-5'), 44.0 (C-11), 41.3 (C-9), 38.1 (C-2), 24.7 (C-3'), 23.6 (C-4').

HR-MS (ESI): calc.: $[M+H]^+$ = 562.1159 amu found: 562.1162 amu.
calc.: $[M+Na]^+$ = 584.0978 amu found: 584.0975 amu.

FT-IR: (ATR) ṽ [cm^{-1}] = 3284 (w), 3059 (w), 3002 (w), 2933 (w), 2851 (w), 2252 (w), 2041 (s), 1960 (s), 1755 (m), 1643 (s), 1608 (m), 1517 (s), 1493 (m), 1453 (s), 1426 (m), 1400 (w), 1318 (m), 1274 (s), 1209 (s), 1166 (s), 1115 (s), 1072 (m), 1022 (m), 995 (m), 908 (m), 861 (w), 806 (w), 760 (w), 727 (s), 695 (m), 646 (w), 612 (s).

2.2. Synthesis of AT-CORM 2-B

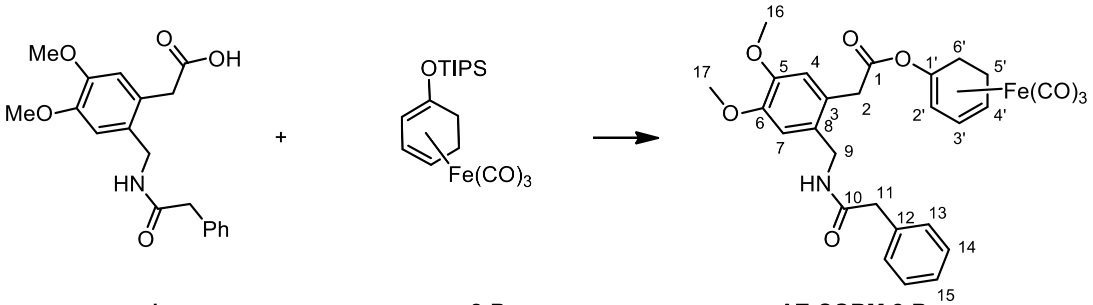

1 *rac*-**2-B** **AT-CORM 2-B**

Under argon atmosphere, a solution of TIPS-protected complex *rac*-**2-B**[1](250 mg, 0.64 mmol, 1.0 eq.) in anhydrous DMF (5.0 mL) was cooled to 0 °C. TBAF (1 M solution in THF, 640 µL, 0.64 mmol, 1.0 eq.) was added and the reaction mixture was stirred for 10 min at 0 °C. Then pyridine (83 µL, 0.638 mmol, 1.0 eq.) and HBF_4*Et_2O (87 µL, 0.638 mmol, 1.0 eq.) were added and the mixture was stirred for further 10 min.

In a second flask, under argon atmosphere, a solution of acid **1**[2] (219 mg, 0.638 mmol, 1.0 eq.) in anhydrous DMF (5 mL) was cooled to 0 °C. Then pyridine (166 µL, 1.28 mmol, 2.0 eq.), DIC (298 µL, 1.91 mmol, 3.0 eq.), HOBt (25.8 mg, 0.191 mmol, 0.3 eq.) and DMAP (23.3 mg, 0.191 mmol, 0.3 eq.) were added. Subsequently, the dienol-complex was added, and the mixture was stirred at room temperature for 12 h.

The mixture was diluted with EtOAc (45 mL), washed with HCl (0.1 N, 2 x 20 mL), water (20 mL) and brine (20 mL). The organic phase was dried with $MgSO_4$ and the solvent was removed under

reduced pressure. The residue was purified by column chromatography (SiO_2, cHex/EtOAc/$CHCl_3$ = 1:1:0.5) to yield **AT-CORM 2-B** (260 mg, 0.463 mmol, 73%) as a yellow oil.

**M($C_{28}H_{27}FeNO_8$):**	561.37 g mol^{-1}.
**TLC:**	R_f (cHex/EtOAc/$CHCl_3$ = 1:1:0.5) = 0.33.
**^{1}H NMR:**	(400 MHz, $CDCl_3$) δ = 7.34 – 7.27 (m, 4H, H-13, H-14), 7.27 – 7.19 (m, 1H, H-15), 6.75 (s, 1H, H-7), 6.64 (s, 1H, H-4), 6.29 (s, 1H, NH), 5.38 – 5.32 (m, 1H, H-2'), 5.11 (ddd, ^3J = 6.6, 4.5, ^4J = 1.1 Hz, 1H, H-3'), 4.47 – 4.23 (m, 2H, H-9), 3.82 (s, 3H, H-16), 3.79 (s, 3H, H-17), 3.59 (s, 2H, H-2), 3.57 (s, 2H, H-11), 3.11 (dddd, ^3J = 6.0, 3.7, ^4J = 2.3, 1.5 Hz, 1H, H-4'), 2.19 – 2.07 (m, 1H, H-6'), 1.98 – 1.81 (m, 1H, H-5'), 1.78 – 1.59 (m, 2H, H-5', H-6').
**^{13}C NMR:**	(100 MHz, $CDCl_3$): δ = 211.2 ($Fe(CO)_3$), 170.7 (C-10, C-1), 148.6 (C-6), 148.5 (C-5), 135.3 (C-12), 129.5 (C-8), 129.4 (C-13), 128.9 (C-14), 127.2 (C-15), 124.2 (C-3), 113.6 (C-4), 112.9 (C-7), 103.5 (C-1'), 81.0 (C-3'), 80.1 (C-2'), 60.7 (C-4'), 56.0 (C-16), 56.0 (C-17), 44.0 (C-11), 41.3 (C-9), 38.3 (C-2), 26.7 (C-6'), 24.1 (C-5').
**HR-MS (ESI):**	calc.: $[M+H]^+$ = 562.1159 amu found: 562.1162 amu.<br>calc.: $[M+Na]^+$ = 584.0978 amu found: 584.0973 amu.
**FT-IR:**	(ATR) $\tilde{\upsilon}$ [cm^{-1}] = 3288 (w), 3059 (w), 3002 (w), 2935 (w), 2909 (w), 2852 (w), 2248 (w), 2042 (s), 1958 (s), 1743 (m), 1643 (s), 1608 (m), 1516 (s), 1493 (m), 1462 (s),1452 (s), 1423 (m), 1325 (m), 1273 (s), 1211 (s), 1180 (s), 1129 (s), 1103 (s), 1029 (m), 1002 (m), 909 (m), 863 (w), 754 (w), 726 (s), 695 (m), 640 (m), 615 (s).

3. NMR Spectra

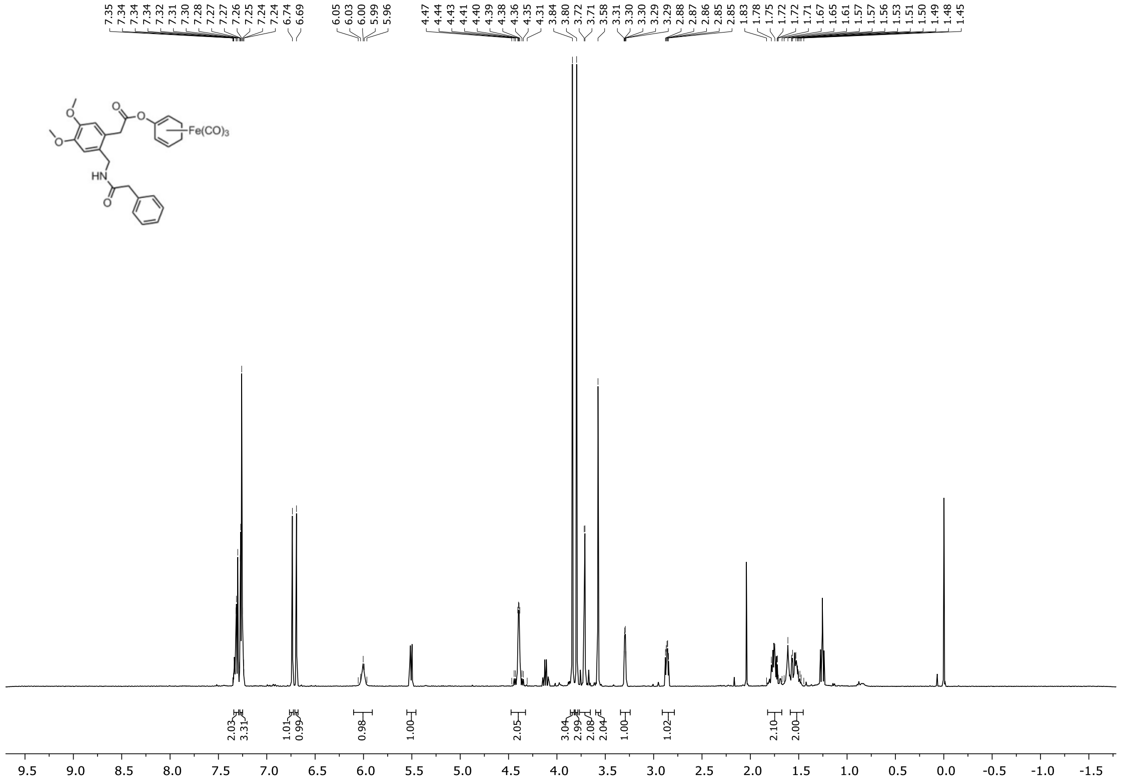

Figure S1: ^{1}H NMR of **AT-CORM 2-A** (400 MHz. $CDCl_3$).

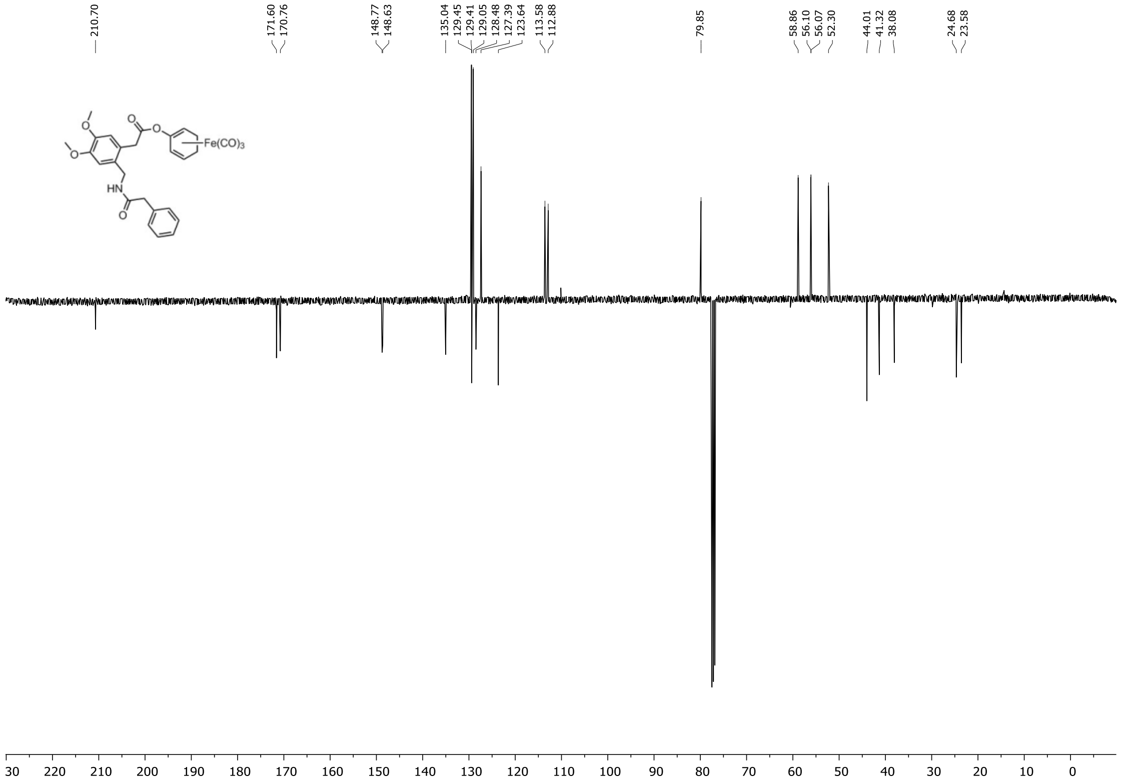

Figure S2: ^{13}C NMR of **AT-CORM 2-A** (100 MHz. $CDCl_3$).

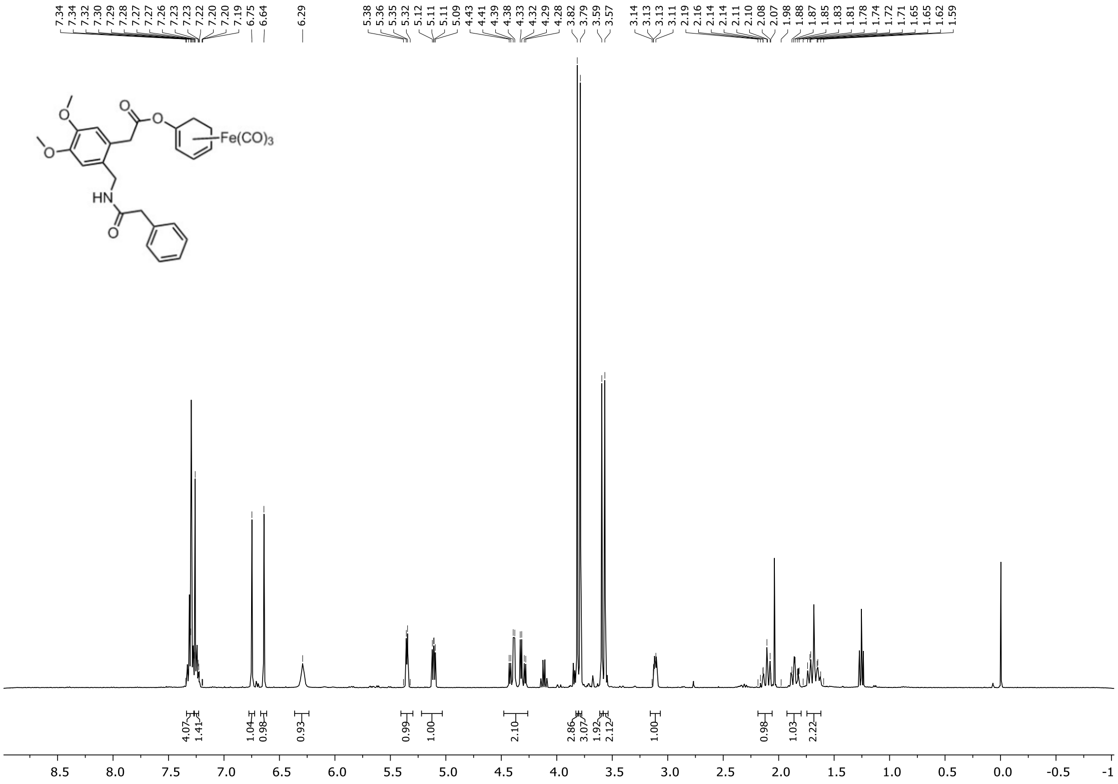

Figure S3: ^{1}H NMR of **AT-CORM 2-B** (400 MHz. $CDCl_3$).

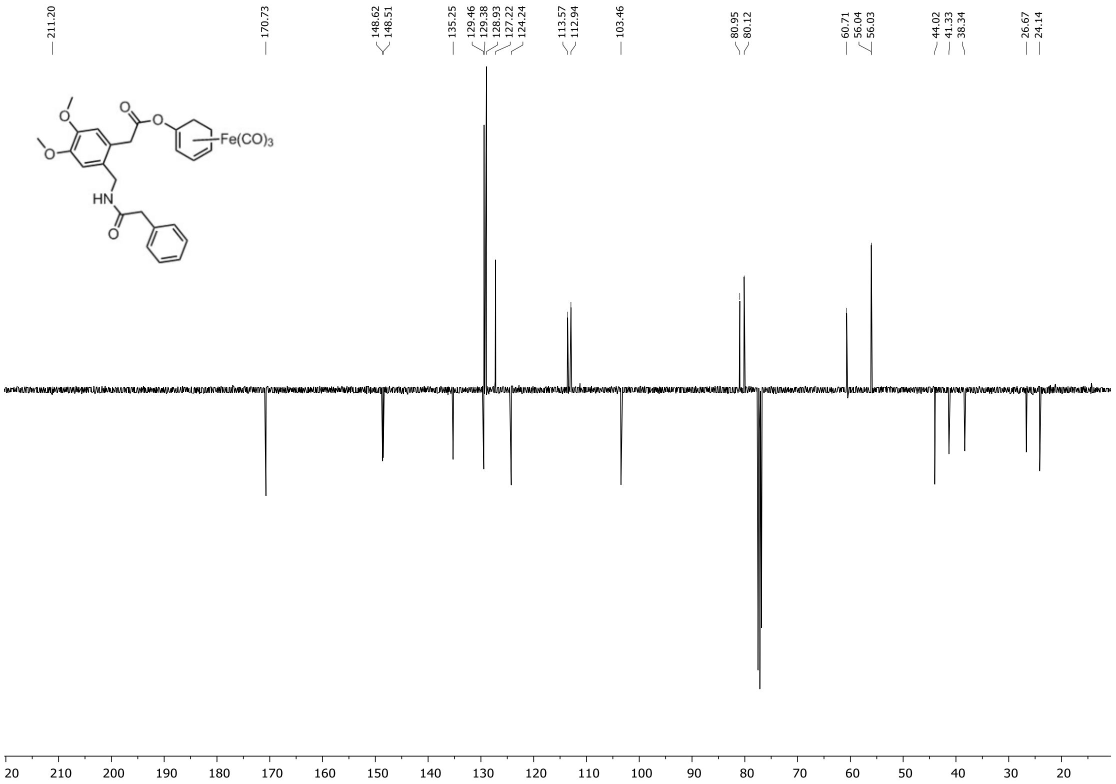

Figure S4: ^{13}C NMR of **AT-CORM 2-B** (100 MHz. $CDCl_3$).

4. In situ quantification of CO and CO_2 release

Headspace GC system description

A *Thermo Scientific* Trace 1300 headspace gas chromatograph equipped with a TriPlus RSH autosampler was used. Detector type: thermal conductivity detector (TCD). Column: Shin carbon ST 100/120 1.0 mm × 2 m 1/16" OD silico. Software: Chromeleon® 7 Data System.

Headspace gas chromatography conditions

Gas-carrier: helium; flow: 15 mL/min; injector temperature: 200 °C; split flow: 150 mL/min; split rate: 10; detector temperature: 200 °C.
Method description: 0 – 2.5 min 35 °C, then to 70 °C with 20 °C/min rate, then 1 min at 70 °C, then to 35 °C with 20 °C/min rate, then 1 min at 35 °C. Injection volume: 50 µL.

Calibration

Eight headspace vials (BGB Analytics, cat. No. 200410-F, 10 mL) were filled with DMSO (0.2 mL) and phosphate buffer (0.1 M, pH = 7.4, 1.0 mL). Subsequently, the vials were closed with gas-tight silicon/PTFE septa crimp caps (BGB Analytics, cat. No. 20030500). Then, a defined gas volume was substituted by CO or CO_2 (0.00 mL, 0.05 mL, 0.10 mL, 0.25 mL, 0.50 mL, 1.00 mL, 1.50 mL, 2.00 mL). After equilibrating the vials for 10 min at 37 °C the composition of the gas phase was determined by headspace GC. These measurements were repeated three times and a calibration curve was generated.

In situ quantification of the CO release properties

The respective complex (36 µmol) was dissolved in DMSO (0.2 mL) and phosphate buffer (0.1 M, pH = 7.4, 1.0 mL) then PLE (15 mg) was added. The mixture was stirred at 37 °C and the amount of released CO assessed through headspace GC using the previously recorded calibration curve. As a control, samples without addition of esterase were analyzed in the same manner.

5. Biological Investigations

Cell culture: Human umbilical vein endothelial cells (HUVEC) were isolated from the fresh human umbilical cords. To this end, a three-way valve was inserted on both ends of the umbilical vein and thoroughly flushed with PBS before 5 ml of collagenase solution (1 mg/ml) (Merck, Darmstadt, Germany) was added. After clamping of the vessel, the umbilical cord was incubated for 10 minutes at 37 °C. Hereafter, the vein was flushed with 15 ml of PBS to collect the endothelial cells. The cells were cultured in 1% gelatin (Fluka, Neu-Ulm, Germany) coated flasks in endothelial cell growth medium (PromoCell, Heidelberg, Germany) supplemented with essential growth factors and 5% fetal bovine serum (Gibco, Carlsbad, USA). Cultures were maintained at 37 °C, 95% relative humidity and 5% CO_2. Confluent monolayers were passaged by TrypLE™ Select Enzyme (Thermo Fisher Scientific, Braunschweig, Germany) and experiments were conducted on cells in passage 2 or 3 at approximately 90-100% confluence. Umbilical cords were obtained from healthy women (Department of Obstetrics, University Medical Center Mannheim) after written informed consent (No. 2015-518N-MA). Isolation was approved by the local ethics committee (Medizinische Ethikkommission II der Medizinischen Fakultät Mannheim)

Cell viability: Cell viability was measured by MTT (3-(4,5-dimethylthiazol-2-yl)-2,5-diphenyltetrazolium)tetrazolium reduction assay. HUVECs (2.5×10^3 cells/well) were seeded on 96-well plates and cultured in normal medium overnight, followed by treatment for 5 h. or overnight with different concentrations of CORMs. Hereafter, MTT (0.5 mg/ml in PBS) was added for 4 h to each well and the plates were incubated at 37 °C, 5% CO_2. Crystals of formazan were dissolved by the addition of solvent solution (4 parts DMSO, 4 parts 10% w/v SDS and 2 parts of PBS/acetic acid in a final concentration of 1.2% v/v) and left for overnight at room temperature. Absorbance was measured with a microplate reader at 560 nm with subtracted background at 670 nm. Relative cell viability was calculated as ratio of OD sample/OD untreated control. For each condition 5 or 6 replicates were used.

Western blotting: For Western blotting, HUVEC were stimulated with TNFα (25 ng/ml) in the presence of various concentrations (0-100 µmol/l) of different CORMs. Cells that were untreated served as control. Supernatants were aspirated, the cells were put on ice and 50 µl of ice-cold lysis buffer (10 mM Tris, PH 7.4, 150 nM NaCl, 5 mM EDTA, 1% Triton X-100, 0.5% Na-Desoxychoalat) supplemented with dithiothreitol (Invitrogen, Carlsbad, USA), protease inhibitor (Roche, Indianapolis, USA), and phosphatase inhibitor (Sigma-Aldrich) was added for 15 minutes. Cell lysates were centrifuged for 10 minutes at 12000 G at 4 °C to remove undissolved cell debris.

Protein concentrations were measured using Bio-Rad Protein Assay Kit. All samples (20 µg of total protein) were diluted in 4x Laemmli sample buffer (Bio-Rad, Hercules, USA) and boiled for 5 minutes to denature proteins. The samples were then loaded on a 10% SDS-PAGE and transferred to a PVDF membrane by semi-dry blotting. Membranes were blocked in TBS (Bio-Rad) containing 0.1% Tween (Sigma-Aldrich) and 5% milk powder for 1 hour at room temperature. Anti-VCAM-1 (R&D Systems, Wiesbaden, Germany) and anti-HO-1 (Enzo, Farmingdale, USA) were used as the first antibody. After incubation with the appropriate horseradish-peroxidase-conjugated secondary antibodies, proteins were visualized by chemiluminescence according to the manufacturer's instructions. Equal protein loading was confirmed by β-actin intensity using specific antibodies (Santa Cruz Biotechnology, Dallas, USA).

RNA isolation and quantitative PCR: Total RNA was prepared using Trizol reagent (Ambion, Carlsbad, USA). RNA quality was confirmed by capillary electrophoresis on an Agilent 2100 bioanalyzer (Agilent Technologies, Palo Alto, CA). All RNA preparations were subjected to treatment with RNase free DNase I (Ambion, Carlsbad, USA) according to the manufacturer's instructions. The expression level of VCAM-1 and HO-1 mRNA was assessed by qPCR. To this end, 1 µg of total RNA was reverse-transcribed into cDNA using the High-Capacity cDNA Reverse Transcription Kit (Applied Biosystems, Foster City, USA). RT reactions were carried out using the following protocol: 16 °C for 30 min, followed by 42°C for 30 min and 85 °C for 5 min in a 2720 Thermal Cycler (Applied Biosystems). Samples were stored at –20 °C until use. Quantitative PCR was performed on a Step-one Plus PCR System (Applied Biosystems) using TaqMan fast advanced master mix (Applied Biosystems) and the following TaqMan probes (Applied Biosystems): VCAM1 (ID: Hs 01003372_m1), HO-1 (HMOX1 ID: Hs01110250_m1) and β-actin (ACTB, ID: Hs 01060665_g1). The following thermal cycling profile was used for all samples: 20 sec at 95 °C followed by 40 cycles of 1 sec at 95 °C and 20 sec at 60 °C. All samples were normalized for equal expression of β-actin. For quantification of mRNA expression, the ΔΔ-Ct-method was used.

6. References

[1] S. Romanski, B. Kraus, M. Guttentag, W. Schlundt, H. Rücker, A. Adler, J.-M. Neudörfl, R. Alberto, S. Amslinger, H.-G. Schmalz, *Dalton Trans.* **2012**, *41*, 13862-13875.

[2] U. Grether, H. Waldmann, *Chem. Eur. J.* **2001**, *7*, 959-971.
